# Nudging Cooperation in a Crowd Experiment

**DOI:** 10.1371/journal.pone.0147125

**Published:** 2016-01-21

**Authors:** Tamara Niella, Nicolás Stier-Moses, Mariano Sigman

**Affiliations:** 1 Universidad Torcuato Di Tella, C1428BIJ, Buenos Aires, Argentina; 2 CONICET, Buenos Aires, Argentina; Middlesex University London, UNITED KINGDOM

## Abstract

We examine the hypothesis that driven by a competition heuristic, people don't even reflect or consider whether a cooperation strategy may be better. As a paradigmatic example of this behavior we propose the zero-sum game fallacy, according to which people believe that resources are fixed even when they are not. We demonstrate that people only cooperate if the competitive heuristic is explicitly overridden in an experiment in which participants play two rounds of a game in which competition is suboptimal. The observed spontaneous behavior for most players was to compete. Then participants were explicitly reminded that the competing strategy may not be optimal. This minor intervention boosted cooperation, implying that competition does not result from lack of trust or willingness to cooperate but instead from the inability to inhibit the competition bias. This activity was performed in a controlled laboratory setting and also as a crowd experiment. Understanding the psychological underpinnings of these behaviors may help us improve cooperation and thus may have vast practical consequences to our society.

## Introduction

In situations that involve scarce resources such as space, time, or goods, people sometimes engage in some form of competitive behavior even when they would be better off if a cooperation agreement could be reached. One important case is the zero-sum (or lump of labor) fallacy, which has been used to study several topics in economic theory, such as poverty, unemployment, retirement, and immigration [1] [2]. The claim is that lack of cooperation may result from the belief that resources are fixed even when they are not. This leads to the potentially misleading conclusion that, in society, if somebody wins somebody else has to lose. In addition, people derive value and pleasure from competition and from beating the rival, even when there are no monetary rewards at stake. When the benefits or cooperation are not evident or explicit, as it happens with the zero-sum fallacy, the tendency to compete may override the possible benefits of cooperation [3].

Another reason why people may be reluctant to cooperate is lack of trust, since this makes one vulnerable to the actions of others. There is a vast tradition of investigating which factors determine whether a person will or will not trust strangers [4], [5], [6], [7], which has relied in classic economic games.

This work examines the hypothesis that driven by a competition heuristic, people do not even reflect or consider whether a cooperation strategy may be better. If the hypothesis is correct, there is a simple way to nudge cooperation: it suffices to make subjects reflect explicitly on whether competition is indeed an optimal strategy.

In the experiments we conducted, we asked participants to repeatedly play the “thumb war game” (TWG), a game in which players score a point when they pin the opponent’s thumb (placing one thumb on top of the other one) to a count of three. We told participants that the goal was to make as many points as possible, which was reinforced by monetary payoffs proportional to the points achieved at the end of the game. We show that participants were driven by the zero-sum fallacy and competed to win the game. Playing to pin the opponent’s thumb takes longer than cooperating, and hence it does not maximize the number of points that both opponents make. The crucial result is that a *nudge* in which we simply reminded participants that a competing strategy may not be optimal boosts cooperation. This suggests that competition does not result from lack of trust or willingness to cooperate but instead from the inability to inhibit the competition bias.

Above and beyond the specific goal of investigating the drive for competition and cooperation, our work has one complementary motivation: to investigate the possibility of obtaining reliable data in a large sample capitalizing on crowd experiments. To this aim, we perform two almost identical experiments: one in a traditional lab settings, and another in a large theater with over one thousand people performing the experiment simultaneously. The latter allows us to obtain very large samples which provide a unique possibility to address a) the issue of reliability which has been a matter of substantial debate in psychological experiments [8] and b) the inference of psychological principles based on a very limited segment of the population [9,10,11,12]

## Materials and Methods

In this section we describe the general aspects of all treatments. Table 1 provides specific details of participants and conditions for each treatment.

**Table 1:** Summary of all treatments’ information about participants and conditions. Includes; purpose of the experiment, number of participants, gender distribution, age range in years, if participants were allowed to discuss a cooperation strategy or not, and what kind of incentives were given in each treatment).

Treatment	Purpose	Place	Nr. of participants	Gender Distribution	Age range years	Where allowed to discuss a cooperation Strategy (Yes/No)	Incentives
**1**	Investigate the tendency to compete even when there are monetary incentives to cooperate and when cooperation strategies cannot be discussed explicitly.	Laboratory	64	W: 35.94% M: 64.06%	18–24	No	Monetary
**2A**	The purpose of this treatment is to collect a large sample to: 1) Identify the effect ofdemographic variables in the tendency to cooperate. 2) Investigate the robustness of laboratory measure in a situation closer to real-life. 3) See if the cooperation ratio remains similar even when there are no monetary rewards.	Theater	850	W: 47% M: 53%	18–32	No	Obtain as many points as possible.
**2B**	To investigate whether allowing participants to talk promotes cooperation.	Theater	690	W: 46% M: 54%	18–32	Yes	Obtain as many points as possible.
**3**	Control treatment to investigate whether change in cooperation in the second round results from instructions or instead from playing the game twice.	Classroom	16	W: 84.62% M: 15.38%	24–62	Yes	Obtain as many points as possible.
**4**	To determine whether after shifting from competition to cooperation participants have an explicit understanding that cooperation results in larger gains.	Classroom	28	W: 28% M: 72%	21–52	Yes	Obtain as many points as possible.

### Participants

#### Experiment 1

64 volunteers (35.94% women, 64.06% men, age: 18–24 years old) participated in a laboratory experiment.

#### Experiment 2

2391 volunteers (1540 players without counting referees) participated in a crowd experiment (46.90% women, 53.10% men, age: 18–32 years old), which was performed in a large theater.

#### Experiment 3

16 volunteers participated in classroom experiment (84.62% women, 15.38% men, age: 24–62 years old).

#### Experiment 4

28 volunteers participated in classroom experiment (28% women, 72% men, age: 21–52 years old).

All participants were native Spanish speakers and all experiments were conducted in Spanish. Consent documentation: Participants provided a verbal consent simply responding to the research assistants the willingness to participate; there is no written consent documentation. Participants did not sign a written consent form due to the brevity of the experiment. Participants were informed that participation in the experiment was completely voluntary and they could simply choose not to participate. Participants were explicitly assured that a) their participation in the experiment was completely voluntary and that they could leave the experiment at any time and b) that all the data was completely anonymous. The consent procedure described here was approved by the Ethics Committee of CEMIC (Comité de Ética de la Dirección de Investigación del Centro de Educación Médica e Investigaciones Clínicas “Norberto Quirno,” Unidad Asociada del CONICET (Protocol # 435)).

### Procedure

In all experiments, participants played two one-minute rounds of sequential TWG. The rules, described in http://en.wikipedia.org/wiki/Thumb_war, are as follows: two players join their right hands together leaving their thumbs up, they greet moving their thumbs left and right three times, and they start playing moving their thumbs to hold one’s thumb on top of the other’s for three seconds. The first one to do it scores a point. Participants were asked to play with their eyes closed.

The instructions given to participants by the experimenter before the first round were: “You are going to play thumb war games for 60 seconds, and each one’s goal is to make as many points as possible. You must play with your eyes closed.”

Between both rounds the experimenter said: “Now you are going to play for another 60 seconds. But first, I want you to remember what your goal was to make as many points as possible. This does NOT imply that you should compete against the other or make more points than your opponent. Actually, there might other ways of playing by which both of you can reach your goal.”

The exact instructions varied for each treatment: in Experiment 1 they included details about monetary rewards, in Experiment 2 whether they were allowed to talk or not and in Experiment 3 there were NO further instructions between rounds. The exact instruction provided for each experiment are presented in Spanish and English in Table 2.

**Table 2:** Summary of instructions given to subjects in each treatment. Both in English (translation) and Spanish (original version).

Treatment	Instructions (English Version)	Original Instructions (Spanish Version)
1	**R1:** You are going to play thumb war games for 60 seconds, and each one’s goal is to make as many points as possible. For each point won, you will get $2 (pesos) at the end of the experiment. You must play with your eyes closed and from now on, you are not allowed to talk to each other.	**R1:** Van a jugar una “pulseada china” por 1 minuto, y el objetivo de cada uno es hacer la mayor cantidad de puntos posibles. Por cada punto ganado, recibirán $2 pesos al final del experimento. Deben jugar con los ojos cerrados y además desde ahora, tienen prohibido hablar entre ustedes.
	**R2:** Ok, now you are going to play for another 60 seconds. But first, I want you to remember what your goal was: to make as many points as possible. This does NOT imply that you should compete against the other or make more points than your opponent. Actually, there might be another way in which both of you can reach your goal.	**R2:** Bien, ahora van a jugar por otros 60 segundos, con las mismas reglas. Pero primero, quiero que recuerden cual era el objetivo de cada uno: hacer la mayor cantidad de puntos posibles. Esto NO implica que deben competir con su compañero o que tienen que hacer mas puntos que el. De hecho, puede que exista otra manera en que ambos logren mejor su objetivo.
**2A** (Oral instructions given by the experimenter)	**R1:** You are going to play for one minute. The goal of each player is to score, in that minute, as many points as possible. The thumb war is played blindly, with eyes closed. Let's start.	**R1:** Van a jugar una “pulseada china” por 1 minuto. El gol de cada jugador es hacer, en ese minuto, la mayor cantidad de puntos posibles. La pulseada se juega a ciegas, con los ojos cerrados. Empecemos.
	**R2:** We will go for another round. But before that I remind you again of the exact instructions I gave you of the game: each player has to try to score as many points as possible. I remind you that this doesn't mean having to score more points than the other player. It doesn't mean that if you lose you have to feel bad or that you have to make more points than the others. Each person has to think how to achieve the goal you have. You may need to collaborate, cooperate, you guess. Let's play again with the goal each one has of trying to score as many points as possible: no matter if everyone scores a lot as well, what you want is to do as many points as possible.	**R2:** Ahora vamos a jugar otra ronda. Pero antes de eso, quiero recordarles nuevamente las instrucciones exactas que les di del juego: cada jugador debe intentar hacer la mayor cantidad de puntos posibles. Les recuerdo que esto no quiere decir que deben hacer mas puntos que el otro jugador. No significa que si pierden tienen que sentirse mal o que tienen que hacer mas puntos que el resto. Cada uno debe pensar como alcanzar su objetivo. Quizás tengan que colaborar, cooperar, ustedes verán.Volvamos a jugar con el objetivo que tiene cada uno de hacer la mayor cantidad de puntos posibles: no importa si el resto también hace muchos puntos, lo que ustedes buscan es hacer la mayor cantidad de puntos posibles.
**2A** (Written instructions given to each participant)	Players are not allowed to talk to each other	Los jugadores no pueden hablar entre ellos.
**2B**	Similar to A without the written instructions forbidding conversation	Similar to A without the written instructions forbidding conversation
**3**	**R1:** You are going to play thumb war games for 60 seconds, and each one’s goal is to make as many points as possible. You must play with your eyes closed.	**R1:** Van a jugar una pulseada china por un minuto, y el objetivo de cada uno es hacer la mayor cantidad de puntos posibles. Deben jugar con los ojos cerrados.
	**R2:** Ok, now you are going to play for another 60 seconds. Each one’s goal is to make as many points as possible. You must play with your eyes closed.	**R2:** Bien, ahora van a jugar por otros 60 segundos. El objetivo de cada uno es hacer la mayor cantidad de puntos posibles. Deben jugar con los ojos cerrados.
**4**	**R1:** You are going to play thumb war games for 60 seconds, and each one’s goal is to make as many points as possible. You must play with your eyes closed.	**R1:** Van a jugar una pulseada china por un minuto, y el objetivo de cada uno es hacer la mayor cantidad de puntos posibles. Deben jugar con los ojos cerrados.
	**R2:** Ok, now you are going to play for another 60 seconds. But first, I want you to remember what your goal was: to make as many points as possible. This does NOT imply that you should compete against the other or make more points than your opponent. Actually, there might be another way in which both of you can reach your goal.	**R2:** Bien, ahora van a jugar por otros 60 segundos, con las mismas reglas. Pero primero, quiero que recuerden cual era el objetivo de cada uno: hacer la mayor cantidad de puntos posibles. Esto NO implica que deben competir con su compañero o que tienen que hacer mas puntos que el. De hecho, puede que exista otra manera en que ambos logren mejor su objetivo.

#### Specific procedures for each experiment

Experiment 1 took place in a lab setting. The experimenter gave the instructions and annotated the results of the game. Participants were asked to not talk during the experiment. This was done, in this specific treatment, to inquire about the emergence of collaboration when there is no opportunity for the players to discuss about specific strategies. Participants in Experiment 1 were paid 2 Argentine pesos (equivalent to approximately 0.25 US dollars as of early 2015) for each point they scored.

Experiment 2 was conducted in a theater, in the context of a TEDx activity (http://tedxriodelaplata.org/tedxperiments/pulseada-china). The experimenter gave the instructions to the audience from the stage. Participants were asked to self organize in groups of three. In each group, two participants were randomly assigned roles of players, while the third participant was assigned the role of “referee.” The referees were given sheets of paper that contained a) the written instructions that complemented the oral instructions provided by the experimenter and b) a form in which they could fill the results of the games played by the players of their group.

The experimenter orally announced the beginning and the end of each round and all groups played simultaneously. After the end of each round, referees had a minute to complete the forms. Figure A in S1 Fig shows the form used to record the results of the experiment. This form was specially designed to allow referees to record rapidly and unambiguously the information of the rounds and to be easily scanned with an automatic procedure as described below.

The information recorded by referees was: (a) participants’ demographic information (age and gender) (b) the results of the all the games of the round up to a maximum of eleven games (c) whether players cooperated and who proposed cooperation, (d) whether someone opened the eyes (even if the instructions asked them not to do so), (e) whether one of the players initiated a conversation to propose cooperation and (f) whether one of the players (or both) broke the cooperation agreement. Referees could also explain how the negotiation took place if there was any.

As we collected a large amount of data in this experiment, we designed the form so that it could be processed using automatic optical mark recognition (OMR). The score sheet had a table of 11 columns and 2 rows with predefined boxes in which the referee marked which player won the games (Figure A in S1 Fig). There was an additional box in which they could handwrite the total number of games won by each player, in case the total number of games exceeded 11. The automatic recognition procedure could not process the total of games won (if they were more than 11) and hence for all analyses in this manuscript we considered results up to the 11th game of each round.

Treatment 2a/2b: Referees were randomly assigned one of two sheets with instructions corresponding to treatment 2a or 2b. The only difference between these treatments was that in 2a, referees were asked to tell the players that they should not talk throughout the game (during rounds or between them). This manipulation was done to investigate the difference in the emergence of cooperation when there is or when there is not opportunity to discuss strategies.

Experiment 3 was conducted in a classroom. The experimenter gave the instructions in front of the class to all participants. Participants were asked to find a partner to play with (the person seating on their back/front). After each round in the experiment, we handed in paper sheets where each participant self-reported their results. The difference of Experiment 3 compared to all other experiments is that subjects were not provided further instructions between the first and second round. This was done to inquire whether a change of strategy between rounds resulted from additional instructions or simply from having played the game for a second time.

Experiment 4 was conducted in a classroom. The setup was almost identical to Experiment 3. The two differences were a) Additional instructions were presented to participants between rounds as in Experiments 1 and 2, and b) After having completed the game, participants who cooperated in round two but not in round one responded a questionnaire explaining the reasons for that change.

### Theoretical Framework

To complement the experiments, we describe a theoretical game that captures the sequence of play of the experiments. This model can be found in Secion 4, “Theoretical Framework”. We characterize the subgame perfect equilibrium of the game, and show that cooperation emerges as a dominant strategy for parameter combinations matching what was empirically observed in the experiments.

## Results

We performed two core experiments (1 and 2) and two control experiments (3 and 4). Experiment 1 took place in a classic lab setting where two participants played TWG against each other while Experiment 2 was performed in a large theater where over one thousand participants played simultaneously. In all experiments, participants played two one-minute rounds that consisted of as many sequential TWG as they could play in this finite time budget. The objective of each player was to maximize the total number of points scored in a round. In Experiment 1, subjects were given a monetary payoff proportional to the amount of points gained, to align incentives with the stated goal. This game is not zero-sum, since the total number of TWGs played in a minute depends on the players’ strategies. Specifically, if the TWG game is played for k rounds, which we refer as TWG(k), the game is zero-sum for all k. However, the key aspect here is that k is not determined in advance because, instead, players are playing for one minute.

By agreeing to play quick games with an alternating winner, both players can obtain more points than when engaged in competition. This is assuming that when players agree to cooperate, irrespective of the actual cooperation pattern, the lack of competition might make the game go faster (hence, they end up playing more rounds in one minute). Whether cooperation leads or not to larger gains depends on parameters of the game (mainly the total time taken by a round when they compete or cooperate as well as the likelihood that each player wins in each game). The parameters in our experiment are such that cooperation is beneficial and competition suboptimal. Please refer to the theoretical framework described in Section 4 for more details.

Our aim is to distinguish between three hypotheses:

H1 Collaboration: Players will cooperate in a simple game when cooperation leads to increased gains.
H2 Zero-sum fallacy: Players do not cooperate because they fail to see that the best strategy is to cooperate. Once they receive the nudge that takes them out of the competition heuristic, they shift to cooperation.
H3 Reluctance to cooperate: Players do not want to cooperate because they do not want to do so even when they are aware that cooperation increases payoffs.

The three hypotheses make distinct predictions on the pattern of cooperation in both rounds. H1 predicts cooperation in both rounds, H2 predicts very low levels of cooperation in the first round and a marked increase in cooperation in the second round (when the possible suboptimality of competition is made explicit), and H3 predicts high levels of competition in both rounds.

Experiment 1 was performed in a classic lab setting, which allowed us to verify that both players listened attentively to the instructions. Participants played the round of TWG with their eyes closed and were instructed not to speak. We did this to make Rounds 1 and 2 as similar as possible by restricting the possibility of accumulating shared verbal knowledge throughout the experiment. This also makes collaboration more difficult since participants need to negotiate by implicit actions. Experiment 2 was performed in a large-scale setup at a theater. As a comparative advantage, this allowed us to collect large quantities of data, which could then be used to regress the data to several demographical variables and to investigate the dynamics of negotiation. It also has the advantage of analyzing cooperation in a situation closer to real life in which instructions are expressed clearly but the degree of attention of participants to the instructions vary widely. Additionally, in Experiment 2 we investigated the effect of verbal negotiations.

**Fig 1:**
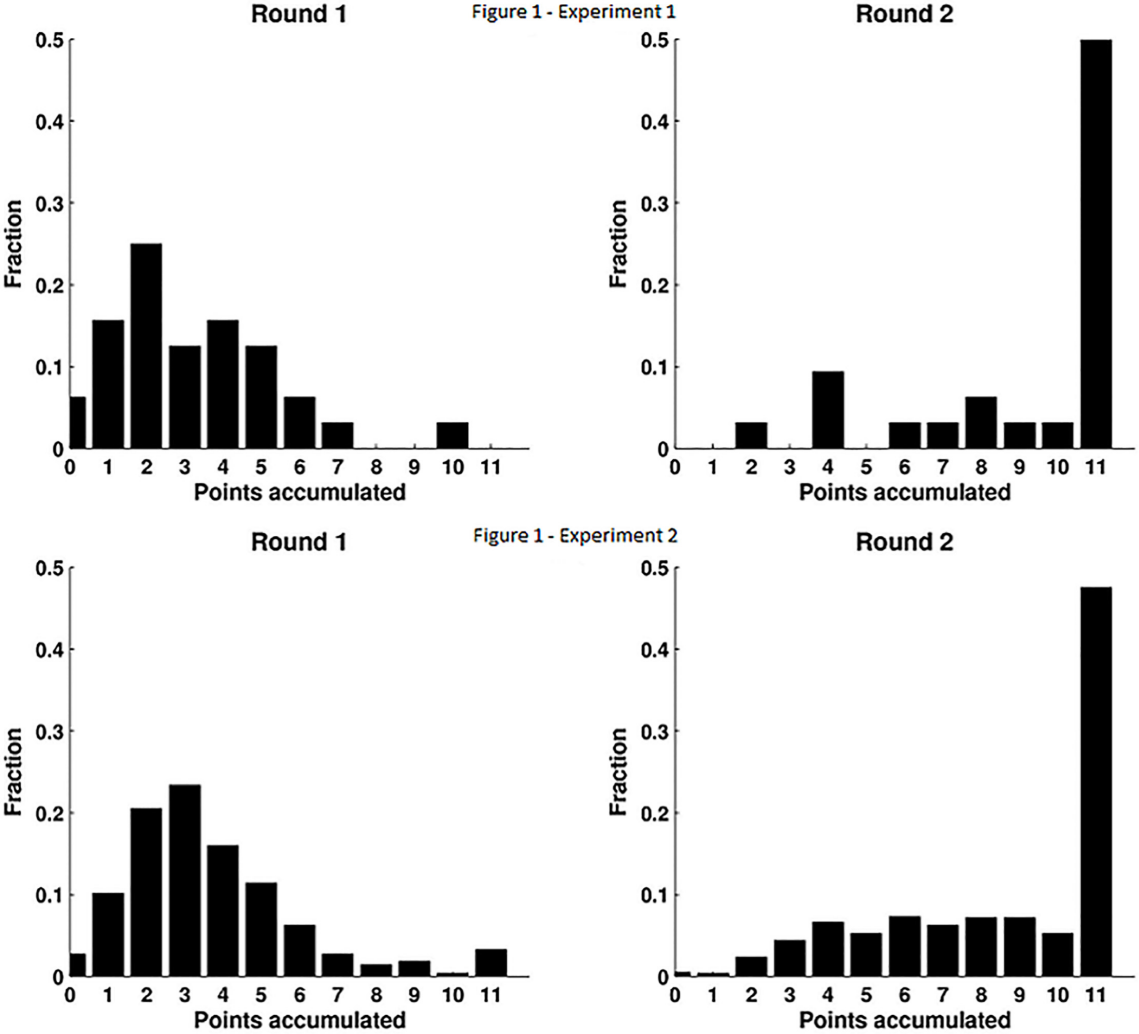
Histograms of accumulated points between both players for the first (left panel) and second (right panel) rounds.

**Fig 2:**
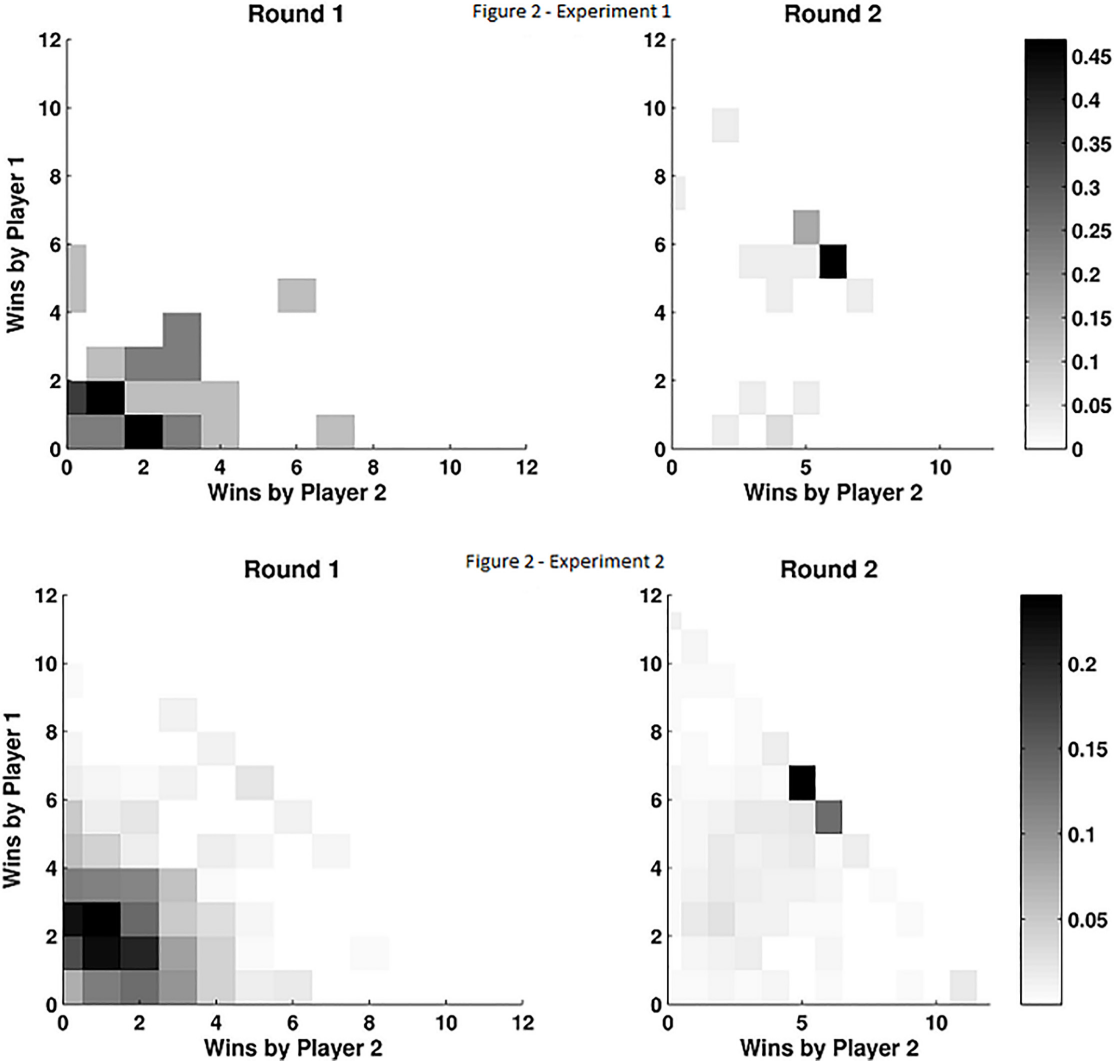
Dimensional histogram of the number of points scored by each player. In the first round (left panel) the distribution is clustered close to the origin showing that players rarely score more than 2 points. In the second round (right panel) the majority of dyads show that both players win 5 or 6 points (11 was the maximal amount of points they could accumulate).

Average scores aggregated within each dyad (Fig 1) and for each individual player (Fig 2) showed an almost three-fold increase in the points obtained in the second round: Experiment 1 (Round 1: 3.1875±0.3874, Round 2: 9.5±0.4709) and Experiment 2 (Round 1: 3.66±0.08, Round 2: 8.54±0.1). A paired t-test revealed that this effect was highly significant (t = 10.35, df = 62, p < 0.001 for Experiment 1; t = 36.2, df = 1538, p < 0.001 for Experiment 2).

The aggregated points in the second round show a very high density of occurrence of 11 points, which was the value at which we saturated the score (68.7% for Experiment 1; 47% for Experiment 2). Instead, the maximum number of points was not attained by any dyad in Experiment 1 and only in 3.2% of the dyads in Experiment 2. The fact that the gain of both players (and not that of one player at the expense of the other) increased in the second round can be seen clearly in the 2-D histogram of paired comparisons (Fig 2). To confirm this statistically we defined min_gain = min (ΔS_1_, ΔS2) for each dyad, where ΔS_i_ indicates the difference in points for player *i* between Round 2 and Round 1. Negative values of ΔS_i_ indicate that player *i* won more points in the first round. Note that if one of the players wins fewer points in Round 2, then min_gain is negative even if this is compensated by an even larger gain of the other player. The average value of min_gain was positive and significantly above zero for both experiments (Experiment 1: 2.125±0.33, t = 6.396, df = 31, p < 0.004; Experiment 2: 1.17±0.07, t = 15.1, df = 769, p < 0.001). This shows that on average, in the second round; even for the player who won the least (or lost the most) the gain is still clearly positive.

**Fig 3:**
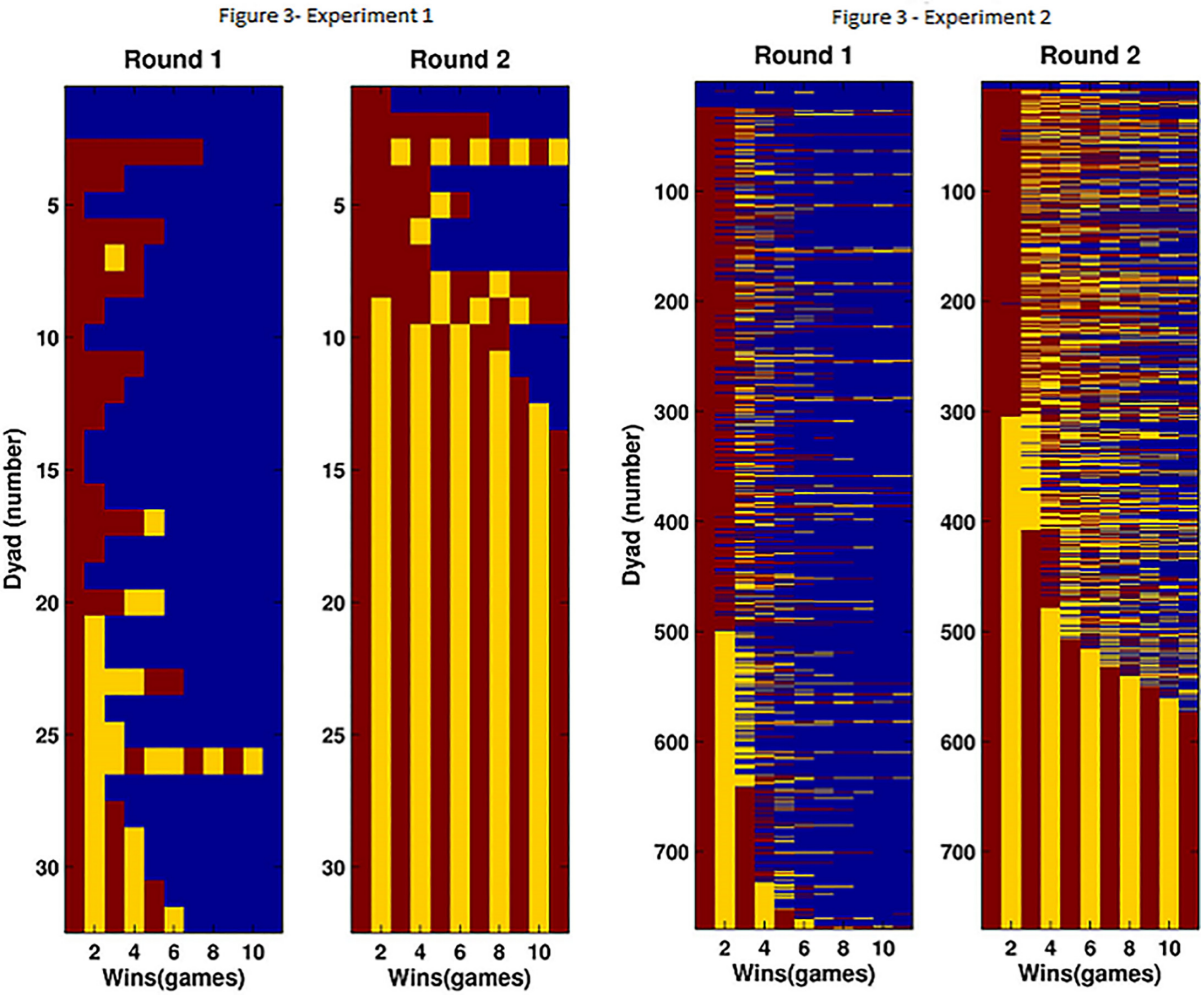
The image depicts a raster of accumulated points by each player. Each line corresponds to a dyad. Within each line columns indicate in color whether the first player (yellow) or the second player (red) won. Player 1 is defined arbitrarily as the one that scores the first point. Data are sorted by sequence proximity to an alternating strategy of cooperation (1-2-1-2-…). Perfect alternating cooperation is achieved by a very high fraction of dyads in the second round (right panels) and with extremely low frequency in the first round (first dyad).

Unfolding the dynamics of each dyad in a raster showing the sequence of games won throughout the minute of play (Fig 3) shows a very clear pattern of cooperation based on an alternation strategy. Note that for participants of Experiment 1 and for half of those of Experiment 2, this strategy is reached without an explicit conversation. To quantify the degree of cooperation, we defined for each dyad a binary value set to 1 if both players obtained at least 4 points and to 0 otherwise. Cooperation, under this definition, was much more frequent in the second round (Experiment 1: 0% in first round and 65.62% in the second round; Experiment 2: 1.55±0.44% in first round and 40.12±1.76% in the second round) and this increase was highly significant (p < 0.001 for Experiment 1; p < 0.001 for Experiment 2) as revealed by a Fisher exact test. This represents an over 30-fold increase in cooperation between the first and second rounds. If we take a stricter definition of cooperation, by considering a dyad to cooperate only when they show alternation of victories to the max number of points, then cooperation in Experiment 1 is still 0% in Round 1 and increases to 59.37% in Round 2. In Experiment 2, it is 0% in Round 1 and increases to 25.7% in Round 2. Note that not even a single one of the 770 recorded dyads achieved this measure of cooperation in the first round.

Hence, Experiments 1 and 2 are consistent with the zero-sum fallacy (Hypothesis H2), and inconsistent with the hypotheses of purely selfish or purely cooperative agents. Note that the fraction of cooperating dyads was larger in Experiment 1 (Experiment 1: 65.62% vs. Experiment 2: 40.12%, p < 0.004), even when considering Experiment 2a and 2b together, regardless of whether participants who were allowed to speak or not, where allowing participants to speak increases the tendency to negotiate. This suggests that a real-life noisy environment where instructions are heard less attentively may strongly affect the range of cooperation and as a consequence the overall gains of the population.

Experiment 2 was based on a large sample (N = 770 dyads) collected using a crowd experiment. This allowed us to investigate some aspects of the cooperation process and demographic variables that affect cooperation. Our analysis led us to the following observations:

1. For the talk-is-permitted treatment (2B) and when there was a negotiation, we recorded whether one of the two players led the negotiation and if that player proposed generous terms (e.g., “first I lose and then I win”, instead of “first I win, then I lose”). In 28% of the dyads one of the two players clearly led the negotiation. Of those dyads, 57.28% of the time, the first game was lost by the player who proposed the negotiation, revealing that most offers were generous (p < 0.015, binomial distribution).
2. We also investigated the probability of cooperation in Round 2 as a function of the results of Round 1. Results showed that those dyads achieving a greater score difference in the first round are less likely to cooperate in the second round than when the first round is more balanced (difference ≥ 3: prob = 0.398, difference < 3: prob = 0.6, Fisher exact comparison p < 0.012).
3. In Experiment 2 we compared the talk-is-permitted (2B) and the talk-is-not-permitted (2A) treatments. We observed over a two-fold increase in cooperation for those dyads that talk throughout the game (2B: prob = 0.697, 2A: prob = 0.269, Fisher exact comparison p < 0.001).
4. There is a significant gender effect in the probability of cooperation in Round 2. Probabilities according to gender are FF = 0.363, MM = 0.647, MF = 0.512, FM = 0.477. Overall, FF dyads show the least likelihood of collaboration (FF vs. MM, p < 0.006; FF vs. FM, p < 0.02; FM vs. MM, p < 0.07). These probabilities are compatible with a very simple model in which the probability of cooperation is ~80% for males and of ~60% for females, and both players are required to be of the cooperator type to establish cooperation.

Finally, we surveyed people’s intuition about cooperation. To this aim, we described the experiment (including a full description of the instructions) to 81 participants (25 had participated in Experiment 2 while the rest had not; both groups show almost identical responses and hence were averaged them together). Results show that people overall overestimate the likelihood of cooperation in Round 1 (29.7%) and understand that there will be more cooperation after emphasizing the non-zero-sum aspect of the game (63.7%). Hence people’s beliefs on how participants would behave reflect an intuition of the zero-sum fallacy but stretch the fact that its effect is underestimated, at least in the context of this game. People believe that about one third of participants will cooperate in the first round while results show that almost none do cooperate, even when it maximizes monetary reward.

H2 hypothesis states that players’ tend to cooperate and earn more points when they are provided a nudge that explicitly makes them reflect on whether competing is the best strategy or not. Here we provided evidence of this by showing that they do not cooperate in the first round, but they do so in the second round. However, it is possible that simply playing the game twice may lead to such understanding without need of explicit instructions. To collect more evidence in favor of H2 we ran two additional experiments.

In Experiment 3 participants played two consecutive rounds, each of one minute, as in Experiments 1 and 2. The difference was that we did not provide further instructions between both rounds. H2 predicts that without explicit instructions that may shift the frame to a more cooperating focus, players will still fail to cooperate. In agreement with this hypothesis, results show that that without further instructions, the number of wins were equal in Round 1 (2.875±0.6382) and Round 2 (2.687±0.4719), t = -0.2362, df = 30, p = 0.8149. This shows that the extra experience of playing again does not help players understand that an alternation strategy may lead to larger payoffs. This provides evidence that a signal to raise their attention to the fact that competition does not optimize their wins is key to override the fallacy.

In Experiment 4 we sought to inquire about participant’s understanding of why they shift strategies between Rounds 1 and 2. This allowed us to determine whether there was an explicit understanding that alternating strategies may lead to larger gains. All the responses provided by participants were classified depending on whether they made reference to maximization of gains and to cooperation (note that the explanation may refer to both, one of them or none.). First we verify that, as in Experiments 1 and 2, gains by each player increased significantly in the second round (Round 1: 1.3571±0.2780, Round 2: 6.4286±1.4973, t = 3.33, df = 54, p = 0.0016), with a 75% of the dyads, switching to a cooperation strategy after further instructions were given between both rounds. Next, for the dyads that did cooperate we analyze the verbal reports (Table 3) indicating why they switched strategies: 38.1% of the explanations made reference to cooperation and 71.43% to maximization of points. Hence, a significant number of participants explicitly understood the reasons that prompted them to change, although a majority describes it as maximizing their gains without explicitly mentioning cooperation.

**Table 3:** Classification of responses from players in Experiment 4 to the question of why they had changed strategies in the second round. Column 1 includes the answer to why they changed strategies. Column 2 is for whether they made reference to cooperation (1 indicates yes, 0 indicates no) and Column 3 is identical but for maximization of points.

Why did you change your strategy in the second round?	Cooperation	Maximization of points
I lost incentives to compete and this way we could win one at a time	1	0
I lost incentives to compete and it was better to cooperate	1	0
Because in the first round I did not think I could get more points through cooperation. When I was told that the goal was simply to get points I did cooperate.	1	0
To get the highest amount of points	0	1
Because I sensed how my partner was playing	1	0
Because it is not possible to maximize both players’ utility if an agreement is not reached	1	1
In the 2nd round we did as much "wins" as possible	0	1
We changed strategy to reach the highest total score	0	1
Because the goal was to maximize the amount of points	0	1
To maximize the amount of points	0	1
I realized that the difference of points between players did not matter but the maximization of the score for both	1	1
The get the highest amount of points possible	0	1
By alternating the wins we could get the highest amount of points in the given time	1	1
It was suggested that there may be a better strategy	0	0
Because the assignment was to obtain the highest amount of points	0	1
Because the goal was to get more points	0	1
By changing the strategy we could both get the highest score	0	1
To reduce the amount of losses	0	1
Because the assignment was that we both should maximize the goal	1	1
Because I lost	0	0

## Theoretical Framework

In this section, we describe a theoretical model that captures the most significant aspects of the experiment presented in the paper. The purpose of this model is two fold: first, provide evidence of how cooperation can be established in the equilibrium of a game that captures the most important elements of the experiment that consisted in playing a series of TWG for 1 minute. Recall that we refer to the 1-minute series of games as a “round,” and to each individual TWG as a “game.” Second we run simulations to show that, within the parameter range in which subjects are playing (typical times for cooperation or competing strategies), cooperation is most often the strategy that results in higher payoffs.

As an abstraction of the main strategic decision of players, at the beginning of the round, players choose whether to compete or to cooperate. During the round they can change their mind, which typically happens when they initially decide to cooperate and then things do not go as planned for the player with the consequence that they start competing as a punishment. At games when players compete, they try to win games at all cost. Instead, when they cooperate they make a tacit agreement to win and concede successive games in a way that can be beneficial to both parties. The compete/cooperate decision represents a component of the strategy space needed to support an equilibrium in which players alternate winning and losing, which is the main insight arising from the experiment we performed. Below we discuss why the cooperation agreement takes that particular form at equilibrium.

We first define the game that conceptualizes the behavior of players. A round is modeled as a series of identical games, played until the time is up. After players decide if they are going to cooperate or to compete, the actual action taken by players is to either try to win or to concede each game. Hence, we describe the outcome of each individual game in terms of those two possibilities. The following matrix lists the probabilities of winning for player 1, the per-game utility for both players, and the duration of the game, as a function of the selected action. Here, p is the probability that player 1 wins, which depends on the skill and aggressiveness of each player.

Table 4 shows expected utilities arising from the Bernoulli random variables that capture the process of winning a game. Since each game is constant-sum, both utilities always sum up to 1. The key aspect of our model is that the duration of each game is given by normal random variables with means and standard deviations that depend on whether competition is established or not (μ1>μ2). When players take the same action, competition arises and the expected game duration is large. Instead, when players take opposite actions, it is clear who will win and hence the game finishes promptly. Note that μ1 and σ1 may depend on p since very unbalanced opponents can be expected to end each game faster than those with similar skills. In any case, for the purpose of the analysis we do here, we can consider that p, μ1, and σ1 are constants.

**Table 4:** Winning probabilities, player utilities and duration of each TWG.

Player 1 \ Player 2		Win	Lose
**Win**	Probability	p	1
	Utility	p, 1-p	1, 0
	Time	N(μ1,σ1)	N(μ2,σ2)
**Lose**	Probability	0	P
	Utility	0, 1	p, 1-p
	Time	N(μ2,σ2)	N(μ1,σ1)

The player utility for the full round is the sum of the individual utilities in the games that were played. First, let us discuss what happens when both players choose to compete for the duration of the round (the case when just one player chooses to compete is similar). In that case, each game will be represented by the win-win entry in Table 4, which results in games taking a long time. Consequently, rounds will consist of a small number of games. The total utility of player 1 can be represented by the number of heads when flipping N1 coins whose head probability is p, where N1 represents the expected number of games in the round and is approximately equal to 60 sec / μ1. Conditionally on both players selecting the compete strategy, the game is constant-sum since a utility of N1 wins must be distributed between both players.

Second, we discuss the situation when both players cooperate throughout the round. We make the assumption that players expect to win approximately half of the time when cooperating. This is a reasonable assumption since players do not know each other before the round and hence they do not have strong priors regarding who the stronger or more skilled player is. Creating a cooperation strategy that achieves utilities for both players proportional to their winning probabilities (p, 1-p) would be hard for the players of the experiment since (a) rounds are not sufficiently long to represent arbitrary probabilities p, (b) players do not know p in advance, and (c) players are not given sufficient time to realize what the optimal strategy is. Splitting the total number of games in half is thus assumed to be a fair outcome given the situation.

There are many ways to split the total number of points in a round in halves. We now argue that alternating winning and losing is the pattern to be expected. While repeatedly playing the games of the 1-minute round, players are unlikely to keep track of time perfectly. In the experiments we conducted, players seemed to be completely focused in playing the games, and there was not a clock anywhere in the room to quickly glance at the time. Hence, it is reasonable to assume that players are not perfectly informed about when the round is going to end. As a consequence, the most reasonable way to cooperate is to alternate winning and conceding games. This is robust since at any moment during the round the difference in total utility is less than one. Of course, there are other ways to cooperate but since other alternatives are more cumbersome they are unlikely to appear in practice (and we haven’t observed them in our experiments). As a result of the earlier discussion, we make the simplifying assumption that the cooperation strategy is based on an alternation of winning and losing for each player. When both players compete, the number of games played in a minute N2 is approximately 60 sec / μ2, defined analogously as N1 but with respect to the random variable N(μ2,σ2). The total expected utility for each player is approximately N2/2.

It is important to highlight that the feature that enables cooperation at equilibrium is the fact that players do not know when the game ends. Without randomness, the cooperation strategy would unravel into competition since in the last game, the player supposed to lose would have an incentive to compete anyway since she has nothing to lose. Then, in the previous-to-last game, the player supposed to lose would anticipate that and try to win, and so on. In our case, when players cooperate, each game is very short compared to the duration of the round (a few seconds vs. a minute). Note that both players are continuously updating the probabilities that the game is going to end as time goes by but they are not precise enough to compute on whose turn the end is going to happen. For this reason, the round can finish with nearly 50% probability when it is anybody’s turn to win, and, consequently, the previous strategic reasoning does not apply, enabling cooperation at equilibrium.

We have already argued that when cooperation is mutually selected, it is optimal for both players to alternate winning and losing, assuming that they switch to the competitive strategy immediately if anybody deviates from the prescribed behavior. Let us now see that cooperation defined in this way is a subgame perfect equilibrium. Representing the initial compete-or-cooperate decision as a 2 by 2 normal-form game and already incorporating the ensuing consequences of each strategic decision, we have the following outcome matrix.

Comparing both strategies from the perspective of player 1, the total utility is p·N1 when competing irrespective of the strategy of player 2. Instead, if player 2 cooperates, the utility of player 1 is N2/2, or the same utility as before if player 2 competes.

We make the assumption that μ1 > 2 μ2 p. It holds when the time spent playing a game with a pre-agreed result is shorter than one played competitively, and when the probability that each player wins is close to 0.5. Actually, for values of p close to 0.5, the inequality holds automatically because μ1 > μ2. In practice, as we illustrate below, this inequality holds for the values derived empirically from the experiments. Under the assumption, cooperation is a (weakly) dominant strategy since the utilities corresponding to it are larger element-wise than those corresponding to competition. Because this applies to both players and since the inequality is strict, cooperation is mutually a best response to the strategy of the other player, making cooperation emerge as the unique Nash-equilibrium of the game.

In summary, the conclusion of the previous theoretical analysis is that irrespective of what the other player does, a player is (weakly) better off by selecting the cooperation strategy, which in the second phase consists of trying to win and concede games alternatively. If at any point in time a player does not behave according to the same strategy, the other player will defect to trying to win at all costs from there on. The main assumptions we have made are that players cannot accurately determine if the game is going to end during the turn they are supposed to win or lose, and that μ1 > 2 μ2 p. The theoretical result supporting cooperation at equilibrium provides evidence for the empirical finding of our experiments.

One last point we want to highlight is that cooperation may create value, which is the driver of the zero-sum fallacy. Indeed, summing the utility of both players in Table 5, we get that the total utility is N1 if one player competes but it is N2 if both cooperate. This clearly illustrates that the game is not constant-sum.

**Table 5:** Anticipated total utilities when players select a strategy for the round.

Player 1 \ Player 2	Compete	Cooperate
**Compete**	p N1, (1-p) N1	p N1, (1-p) N1
**Cooperate**	p N1, (1-p) N1	N2/2, N2/2

**Fig 4:**
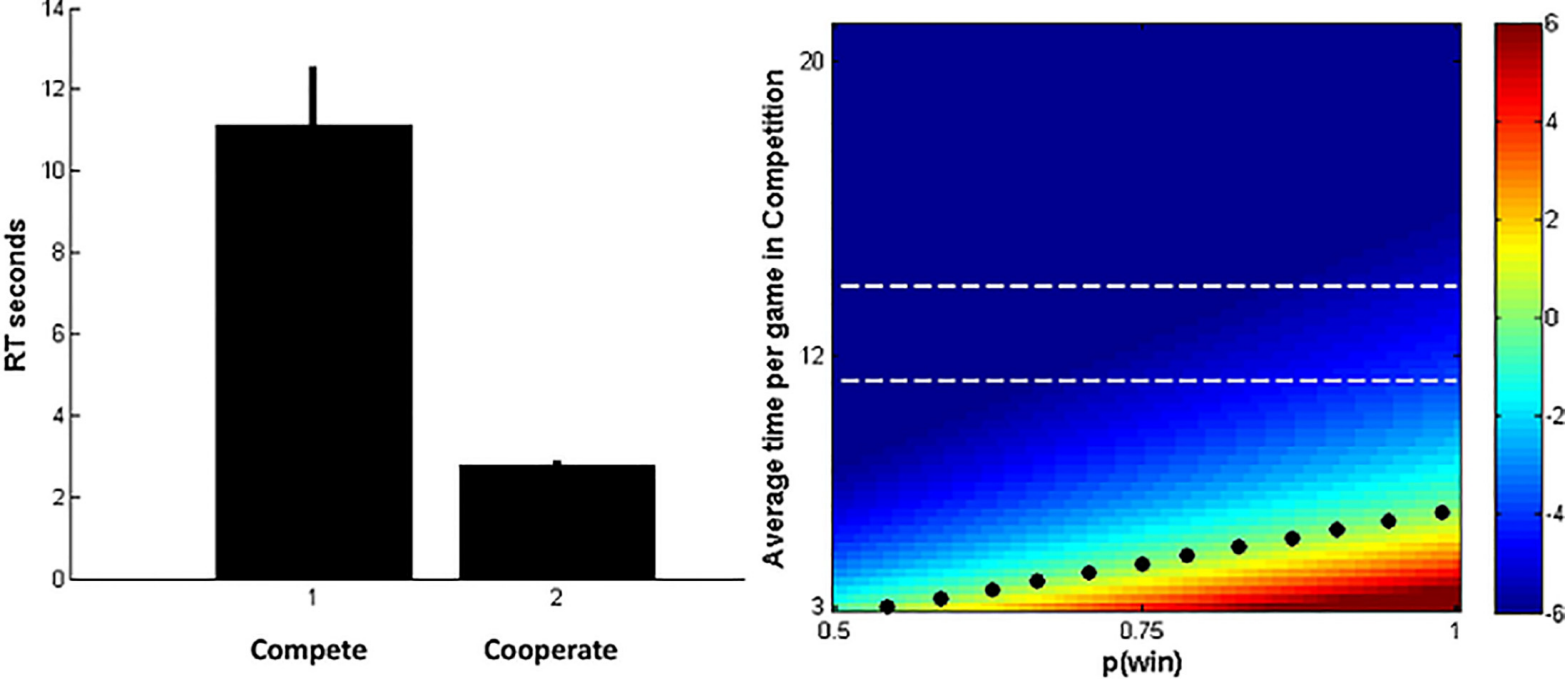
Distribution of the duration of the game under competition and cooperation (left panel), and excess games that can be won by competition with respect to cooperation (right panel).

Finally, to illustrate the theoretical analysis and the conditions under which it is advantageous to cooperate, we plot payoffs according to each strategy as a function of parameters estimated from the experiments. This allows us to validate that the experiments agree with the predictions of the theoretical model. Under cooperation, we observed that the typical time of a game is about 2.6 seconds since opponents just need to agree to pin the thumb. There may be some initial time to set up the strategy, but even when this time is taken into account, the resulting duration is less than 4 seconds. Instead, under competition, we observed empirically that the average duration of a game is about 15 seconds. We illustrate the distributions of duration under both strategies in the left panel of Fig 4.

On the right panel of Fig 4, we indicate which strategy is more convenient as a function of the probability of winning a game (x axis) and the average duration of a game when competing (y axis). For the true average duration under a cooperation strategy, the plot depicts the difference between the games won under competition and those won under cooperation. We can see how the optimal strategy depends on the parameters. When games played competitively are short or when one player is much stronger than the opponent, the strong player prefers to compete (lower right area of the plot with positive numbers). Instead, for longer games or for more balanced players, cooperation is preferable (upper left area of plot with negative numbers). The dotted line starting at the origin represents combination of parameters for which players are indifferent between both strategies. For the band between the horizontal dotted lines, which represents the durations we observed in practice, the dominant strategy is cooperation. This validates our experiments and provides further evidence that our hypothesis holds.

## Discussion

Our results provide strong evidence that people tend to compete albeit it is in their best interest to cooperate. More importantly, we show that sub-optimal competition may result from a lack of reflection on the instructions (even when they are expressed precisely and with full attention of the players as in Experiment 1) since participants changed their behavior once the non zero-sum aspect of the game was stressed. Competition may arise because of prior experience playing this or other games, which may cue players to compete regardless of what the instructions seem to indicate. While here we played a non-zero-sum version of TWG by playing as many games as possible in one minute, the more traditional version (usually played in a best-of-three format) is a zero-sum game. Hence it is likely that subjects will not pay attention to this fact because they have the strong prior that this is a competition game. This could be understood as a form of priming or inductive reasoning, where people project information from known cases to the unknown [13,14]; or even of social influence [15]. A key aspect of our findings is that a simple nudge, by just having people reflect on whether competition is the optimal strategy to achieve their goal, promotes cooperation.

Competition causes pleasure when playing [16]. This was clear in our experiment; a simple observation of the videos revealed that participants smiled much more during the first round than during the second one. Thus it is also possible that people may be unwilling to cooperate because the game becomes more boring. Similarly, participants may have a prior that making an agreement to alternatively win and concede games may be some form of cheating. While these motivations to avoid cooperation are possible, the most important fact is that when they receive a nudge which questions about the optimality of competition, they almost always switch to the strategy of alternating the winner. Hence people’s reluctance to collaborate cannot be explained by the no-fun or alternating-wins-is-cheating hypotheses. These possible behaviors may dominate only when the zero-sum fallacy prevents participants from clearly understanding that cooperation is Pareto-dominant. This shows that the zero-sum fallacy plays a significant role, above and beyond the effect of trust in people´s willingness to cooperate. It is possible that the fraction that still does not cooperate may be due to the fact of the displeasure of losing.

What determines whether a player will or will not cooperate?. Of course answering such a general question is beyond the reach of this manuscript but we hint several ideas. One relates to trust, and as we stated in the introduction, has been the focus of ample work [4,5,6,7,17,18]. Here we present another idea, which is that players may fail to understand the benefit of cooperation. In our case, this comes from a strong prior. In the classic way of playing TWG there is no gain in cooperating. Hence, just stating the objectives of the game does not suffice to override a strong competition heuristic. Re-emphasizing the possible benefits of cooperation (even without making them explicit) helps players understand that cooperation may lead to larger gains.

This result is reminiscent of investigations of framing effects (e.g., the work of Brañas [19] on dictator games) and social value orientations (SVO) introduced by Messick and McClintock [20] and McClintock [21]. The notion of SVO brings up the fact that people are not always selfishly motivated to maximize their own payoffs. A person’s SVO represent her preferences regarding the allocation of resources such as money between themselves and others. Today, SVO is usually studied as a stable individual trait [22,23] with individualistic, cooperative (maximizing the payoff to both players), and competitive (maximizing the difference between own and other’s payoffs) orientations. As a consequence, altruism (maximizing other’s payoffs), equality seeking (minimizing the difference between own and other’s payoffs) ended up as part of it.

In an important study related to our work, Messick and McClintock [20] manipulated the willingness to cooperate in the following way: the other player was referred to either as an “opponent” (to induce a competitive SVO) or as a “partner” (to induce a cooperative SVO). The authors also displayed the players’ accumulated scores in different ways to draw attention to own payoffs, joint payoffs, or relative payoffs. More recent researchers have manipulated SVO in other ways, and Abbink and Henning-Schmidt [24] commented that experimental comparisons of neutral and suggestively framed games are surprisingly rare, but Yamagishi et al. [25], Eriksson and Strimling [26], and others have reported effects. This is all in fact very close to our research, where we state the TWG being a competitive game as the frame, and that this can only be changed with an explicit instruction which can override the competitive frame.

We observed an intriguing gender difference in the number of dyads that cooperated. Overall, women were more inclined to compete. Other studies have consistently observed that gender is an important factor of economic and strategic behavior [18]. Overall, it has been found that in decisions without risk, such as a dictator game in which the player freely decides how to split an endowment [27], women’s behavior is more altruistic. Instead, in decisions under risk these differences vanish [18]. Another finding is the effect of chivalry; for instance, men accept lower offers from women than for men in the ultimatum game [28]. Our results indicating that women compete more are intriguing and somehow distinct from previous findings in economic games. This certainly requires much more detailed investigation to discard two alternative explanations: either the internalization of the zero-sum fallacy depends on gender or, it may be that even after understanding the fallacy, women are more likely to persist in competing behavior for the reasons outlined above (motivation of competition, feeling that cooperating is some kind of cheating, etc.).

There was a difference in the degree of cooperation between Experiments 1 and 2, which may be explained by at least three different alternatives that cannot be distinguished from our previous data: (a) Experiment 1 had monetary rewards. It is possible that when players understand that cooperation is optimal, payoffs will motivate players to cooperate against all other reasons to not cooperate (selfish desire to win, the fun of competition, the view that games should not be arranged). Without payoffs, players may be more motivated to compete and play than earning more points [29], [30]. (b) Players in Experiment 1 were more likely to know each other (because they belonged to the same university campus) and thus it is possible that they are more willing to cooperate (and yet rarely do so in the first round), and (c) Instructions were delivered privately and in a quiet room in Experiment 1 and, instead, to a massive audience in a noisy environment in Experiment 2. Hence it is likely that many of the players in Experiment 2 did not attentively listen to the instructions and hence missed the non-zero aspect of the game. Of these alternatives we were able to discard Hypothesis 1 by performing an experiment analogous to Experiment 1 but without payoffs. Results show that the degree of cooperation did not differ significantly in both treatments (60% without payoffs, 68.18% with payoffs, Fisher exact comparison p = 0.7026).

We saw that the likelihood of cooperation in the second round depends on the imbalance of players’ skills and strengths in the first round. A stronger player in the first round is less likely to enter the cooperation agreement and will push on with competition. This can be rationalized to indicate that this player does not need help from the other player to win as many points. Nevertheless, we did not see that these players ended up making more points than those that cooperated because every game was slower. Another factor that affected cooperation was whether participants could talk or not in Experiment 2. Being allowed to talk increases the chances of cooperation, which could be explained by the fact that one of the players is more likely to quickly propose an agreement. An alternative explanation is that being allowed to talk can induce players to know more about each other and hence creates empathy. In those circumstances players may find it harder to compete.

One specific benefit of our experimental procedure is that it involves a large sample that is more heterogeneous than those in traditional laboratory experiments [8,12]. It has been discussed that many of the conclusions obtained in psychological experiments reflect aspects of the very specific population of undergraduate students [9,10,11]. Our work relies on a crowd experiment that shows that these conclusions are not specific to a relatively narrow segment of the population. The participants in this activity were selected at random from a larger set of 30.000 applicants to TEDxRiodelaPlata (http://tedxriodelaplata.org/) which include people with diverse educational backgrounds, professions, and ages. While we did not measure Socio Economic Status or education background in our sample, simply looking at the age distribution, it is clear that the heterogeneity of our sample is much wider than a group of college students.

Our work also contributes to solve whether it is possible to obtain reliable data in a large sample. The crowd experiment allowed us to obtain a very large sample which provides a unique possibility to address a) the issue of reliability which has been a matter of substantial debate in psychological experiments [8] and b) as discussed above, the inference of psychological principles based on a very limited segment of the population [9,10,11,12]. While there are clear advantages to performing large-scale crowd experiments, there are also some concerns: a) There is little control over whether people follow the instructions and monitoring their behavior throughout the game is difficult. b) The data acquisition also relies on the participants of the experiment. c) The participants’ mindsets could be more focused in the entertainment, possibly diminishing external validity. Determining whether participants in a laboratory are more valid and reliable than in a large audience is in essence an empirical endeavor. While our work cannot respond which observable is closer to real-life decision making, we can shed light on this issue by showing that results on a large audience are reliable and very similar to those obtained in very controlled setups in a laboratory.

Our results provide a first step to develop strategies to override people’s tendency to compete and to nudge the system to an optimal collective behavior. This may have an impact in problems of great economic pertinence in our society. For instance, the situation that led us to conduct the experiments described here was the fact that competition arises when car drivers go through non-signalized intersections. In some societies, all drivers try to go before each other, instead of taking turns as in other societies. This generates additional waiting time arising from suboptimal competition. Since the situation can be mapped to the TWG described in the paper, one may wonder if some type of reminder shown in the intersections can nudge cooperation and reduce driving time.

## Supporting Information

S1 Dataset Data from Online Survey and Experiments 1, 2, 3 and 4. (ZIP)

S1 Fig Fig A. A sample of the form used to record the results of the experiment by the referees in Experiment 2 (A and B). (PDF)
