# Supplementary material for: Nudging Cooperation in a Crowd Experiment
Source: PLoS One. 2016 Jan 21;11(1):e0147125. doi: 10.1371/journal.pone.0147125 (PMC4721918; doi:10.1371/journal.pone.0147125)
Supplement: S1 Fig — (PDF) [file pone.0147125.s002.pdf]

Información Jugadores

Jugador 1		Jugador 2	
Edad<br>○ 10-20 ○ 20-40 ○ 40-60 ○ 60+	Género<br>○ M ○ F	Edad<br>○ 10-20 ○ 20-40 ○ 40-60 ○ 60+	Género<br>○ M ○ F
Email		Email	

Primer set (anotar mientras juegan)

Partidas ganadas

Cada vez que termina una partida (durante las primeras once partidas) anotá quién la ganó en la columna correspondiente. Si se jugaron más de 11 partidas en este set anotá además en el casillero de la derecha cuántas ganó en total cada jugador.

	P. 1	P. 2	P. 3	P. 4	P. 5	P. 6	P. 7	P. 8	P. 9	P. 10	P. 11	Total
Gana Jugador 1	○	○	○	○	○	○	○	○	○	○	○	
Gana Jugador 2	○	○	○	○	○	○	○	○	○	○	○	

Luego del juego

¿Conversaron una negociación? ○ **Sí** ○ **No** - Si la respuesta es “Sí”:

¿Cuándo fue? ○ **Antes de la partida** ○ **En los primeros 10 seg.** ○ **Duró más de 10 seg.**

	¿Espió?	¿Propuso y / o manejó la negociación?	¿Traicionó la negociación?
Jugador 1	○	○	○
Jugador 2	○	○	○
Ambos	○	○	○
Ninguno	○	○	○

Si podés, describí en dos frases la negociación:

A 0001

Tratá de pintar cada casillero (no solo hacer una cruz) para que sea más fácil luego escanear los formularios automáticamente.

¡Gracias!

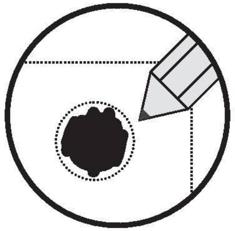

Segundo set (anotar mientras juegan)

Partidas ganadas

Cada vez que termina una partida (durante las primeras once partidas) anotá quién la ganó en la columna correspondiente. Si se jugaron más de 11 partidas en este set anotá además en el casillero de la derecha cuántas ganó en total cada jugador.

	P. 1	P. 2	P. 3	P. 4	P. 5	P. 6	P. 7	P. 8	P. 9	P. 10	P. 11	Total
Gana Jugador 1	○	○	○	○	○	○	○	○	○	○	○	
Gana Jugador 2	○	○	○	○	○	○	○	○	○	○	○	

Luego del juego

¿Conversaron una negociación? ○ **Sí** ○ **No** - Si la respuesta es “Sí”:

¿Cuándo fue? ○ **Antes de la partida** ○ **En los primeros 10 seg.** ○ **Duró más de 10 seg.**

	¿Espió?	¿Propuso y / o manejó la negociación?	¿Traicionó la negociación?
Jugador 1	○	○	○
Jugador 2	○	○	○
Ambos	○	○	○
Ninguno	○	○	○

Si podés, describí en dos frases la negociación:
